# Core promoter information content correlates with optimal growth temperature

**DOI:** 10.1038/s41598-018-19495-8

**Published:** 2018-01-22

**Authors:** Ariel A. Aptekmann, Alejandro D. Nadra

**Affiliations:** 1 Universidad de Buenos Aires, Facultad de Ciencias Exactas y Naturales, Departamento de Química Biológica, Laboratorio de Bioquímica Estructural, Buenos Aires, Argentina; 2 CONICET- Universidad de Buenos Aires, Instituto de Química Biológica de la Facultad de Ciencias Exactas y Naturales (IQUIBICEN). Intendente Güiraldes 2160, C1428EGA Buenos Aires, Argentina; 3 Universidad de Buenos Aires, Facultad de Ciencias Exactas y Naturales, Departamento de Fisiología, Biología Molecular y Celular, Laboratorio de Bioquímica Estructural, Buenos Aires, Argentina

## Abstract

The subtle mechanisms by which protein-DNA interactions remain functional across a wide range of temperatures are largely unknown. In this work, we manually curated available information relating fully sequenced archaeal genomes with organism growth temperatures. We built a motif that represents the core promoter of each species and calculated its information content. We then studied the relation between optimal growth temperature (OGT) and information content (IC) in the promoter region.We found a positive correlation between G + C content and OGT in tRNA regions and not in overall genome. Furthermore, we found that there is a positive correlation between information content and optimal growth temperatures in Archaea. This can’t be explained by an increased C+G composition nor by other obvious mechanisms. These findings suggest that increased information content could produce a positive fitness in organisms living at high temperatures. We suggest that molecular information theory may need to be adapted for hyperthermophiles.

## Introduction

Every organism arises from a similar organism and lives in a physicochemical environment. Thus, every form of life has been constrained by what its genes allow it to be and by the environment in which it develops. Regarding physical and chemical extremes, aside from the need for liquid water, no definite limits have been established for life under extreme conditions^1^. Regarding genetic information an organism evolves by mutations and recombinations based on what was inherited from its ancestors. Adaptations to extreme environments is a complex process and there is scarce information on many of its aspects. Trends in the base composition of sequences across organisms living at different temperatures, allowed researchers to draw some conclusions. As for example the relation between enhanced G + C content with higher optimal growth temperature (OGT)^2^, although there is some controversy on this issue^3^.

We hypothesize that organisms that thrive in extreme environments might have been affected by the selective pressure imposed by this conditions. In particular, sequence composition bias may operate in proteins, DNA and its interaction. To evaluate this hypothesis we looked for relatively narrow natural system with abundant information available. Archaea emerged as excellent candidates since they include the majority of the hyperthermophiles, and there are many described species, living in a wide range of temperatures. Within archaea, there is an essential biological process that has been the subject of several evolutionary and biophysical studies, transcription initiation, where a TATA box Binding Protein (TBP) interacts with its target site. TBP is involved in promoter recognition, the first step of transcription initiation. TBP is universally conserved and essential in archaea and eukaryotes. We expect TBP and TATA box to co-evolve, responding to a number of physicochemical factors like temperature, pressure, salinity, and other environmental conditions. In extremophiles, TBPs have to be stable and to function in species that span an extremely wide range of optimal growth temperatures, from below 0 °C to more than 100 °C. Thus, the archaeal TBP family is ideally suited to study the evolutionary adaptation of a DNA binding protein in a wide range of temperatures. Protein-DNA interactions are central to cell activity regulation. To accomplish its function, a DNA binding protein must locate and bind its target sequence in a huge excess of non specific DNA. Although in biochemical research the target sites were usually represented by one or a few sequences, or by a “consensus sequence”, it is better represented as a sequence logo^4^, relatively tolerant to sequence variations, in contrast to a strict string of definite letters. Furthermore, consensus sequences are frequently misunderstood^5^ and thus, we choose to work with sequence logos to represent sequence diversity in macromolecular interactions. Far from being random, sequence variability is biologically relevant and is related to the underlying process of protein-DNA interaction which can be associated to an information content (IC).

Estimation of a binding site’s information content requires one to know the majority of sites in a genome, as well as its background composition. In the last years, several genomes from archaeal thermophiles have been sequenced and published, making a full genome search for binding site occurrences possible. Care must be taken in order to identify binding sites independently of their sequences, to avoid biasing the motif towards the query. This is possible nowadays for a limited number of binding sites that are highly characterized, quite ubiquitous genome wide and easily identifiable, as the TBS (TBP binding site).

We took advantage of the existence of only one RNA-polimerase (pol II) in archaea, which is TBP dependent^6^ (with the interesting exception of methanoarchaea). As a consequence, all de-novo transcription needs a site for TBP. TBP is a very well studied protein, that binds within a hundred base pairs of the transcription start site^7^. To avoid the additional challenge of identifying ORFs, we focused on a subset of genes of easy and reliable identification: tRNAs. Transcription start sites (TSS) for tRNA, are particularly easily located in a genome using structure based tRNA recognition software^8^. We built a data base of 78 archaeal species ranging from 18 to 100 °C OGT. Interestingly, we found that G + C content increases with OGT in tRNA coding regions but not in promoter regions or complete genomes. For each species we derived its TBP binding motif and calculated its information content. This value was then correlated with the reported OGT, obtaining a positive trend. This correlation can not be explained by G + C content, nor by the other variables we took into account. Furthermore, we suggest that the IC increase with temperature may have a positive fitness. Finally, we suggest that living temperature may affect protein-DNA recognition (either directly or by unknown mediators) and that it may be needed to be explicitly accounted in molecular information theory.

## Results and Discussion

### Genomes

Even though there exist some databases of archaea informing OGT^9,10^, to obtain a high degree of confidence in our data, we decided to curate our dataset by reviewing bibliography and existing databases. In Table S1 we present a collection of data from multiple sources, about archaea with fully sequenced genome and reported optimal growth temperatures. Table 1 reports several parameters for a subset of species analyzed in this work. Considering previous reports^2,3^ we evaluated whether a correlation exists in our dataset regarding genomic G + C content and OGT. While there is a clear increase in 16 S Ribosomal RNA’s G + C content with temperature, a significant correlation was not observed among genome G + C content and OGT (Figure S1). This seems to be coherent with what happens in eubacteria where structured RNA’s G + C content correlates with OGT^11^, and may also be of interest in the long held discussion about whether genomic G + C correlates with OGT^12^ or not^13^.

**Table 1:** Summary of acheal species evaluated informing its optimal growth temperature (OGT), genomic G + C content (G + C), motif’s information content (IC) and motif’s relative entropy (RE).

Index	*NC*_*code*	Name	OGT (°C)	IC (bits)	RE (bits)	G + C (%)
0	*NC*_014297	*Halalkalicoccus jeotgali*	35.5	11.3	13.2	0.65
1	*NC*_015666	*Halopiger xanaduensis*	37	11	13.6	0.66
2	*NC*_019964	*Halovivax ruber*	37	10.8	13	0.64
3	*NC*_020388	*Natronomonas moolapensis*	38.5	12.6	14	0.65
4	*NC*_021592	*Ferroplasma acidarmanus*	38.5	11.4	11.7	0.36
5	*NC*_013922	*Natrialba magadii*	39	13.9	15.9	0.61
6	*NC*_014729	*Halogeometricum borinquense*	41	12.9	14.6	0.61
7	*NC*_013202	*Halomicrobium mukohataei*	42.5	13.3	15.2	0.66
8	*NC*_013967	*Haloferax volcanii*	45	13.3	16.8	0.67
9	*NC*_019974	*Natronococcus occultus*	45	12.9	15.2	0.65
10	*NC*_007426	*Natronomonas pharaonis*	45	10.7	11.9	0.63
11	*NC*_019792	*Natronobacterium gregoryi*	47	11.7	13.9	0.62
12	*NC*_017941	*Haloferax mediterranei*	49	13.8	15.6	0.61
13	*NC*_002607	*Halobacterium salinarum*	49	13.6	16.5	0.68
14	*NC*_006396	*Haloarcula marismortui*	49	11.7	13.4	0.62
15	*NC*_013743	*Haloterrigena turkmenica*	51	12.4	15.2	0.66
16	*NC*_019962	*Natrinema pellirubrum*	51	13.4	16	0.65
17	*NC*_017461	*Fervidicoccus fontis*	67.5	16.9	13.9	0.37
18	*NC*_021169	*Archaeoglobus sulfaticallidus*	75	12.6	12.1	0.43
19	*NC*_000917	*Archaeoglobus fulgidus*	76	12.9	12.6	0.49
20	*NC*_015320	*Archaeoglobus veneficus*	77.5	14	13.4	0.47
21	*NC*_012883	*Thermococcus sibiricus*	78	19.5	17.5	0.4
22	*NC*_015151	*Vulcanisaeta moutnovskia*	79	18.8	17.1	0.42
23	*NC*_013849	*Ferroglobus placidus*	80	13.7	13.4	0.44
24	*NC*_018001	*Desulfurococcus fermentans*	81	15	14.3	0.45
25	*NC*_008698	*Thermofilum pendens*	81.2	14.8	16.7	0.58
26	*NC*_013741	*Archaeoglobus profundus*	82	13.8	12.4	0.42
27	*NC*_014374	*Acidilobus saccharovorans*	82.5	11.9	13.2	0.57
28	*NC*_014804	*Thermococcus barophilus*	85	18.9	17.3	0.42
29	*NC*_014961	*Desulfurococcus mucosus*	85	14.1	14.8	0.53
30	*NC*_022521	*Aeropyrum camini*	85	13.1	13.6	0.57
31	*NC*_014160	*Thermosphaera aggregans*	85	12.8	12.5	0.47
32	*NC*_014537	*Vulcanisaeta distributa*	87.5	18.8	17.7	0.45
33	*NC*_015315	*Thermoproteus uzoniensis*	90	16.5	19.1	0.6
34	*NC*_016885	*Pyrobaculum oguniense*	92	17.5	19	0.55
35	NC_014471	*Ignisphaera aggregans*	93.5	13.1	12.3	0.36
36	*NC*_015680	*Pyrococcus yayanosii*	98	17.9	18.3	0.52
37	*NC*_000868	*Pyrococcus abyssi*	100	17.8	16.7	0.45
38	*NC*_003413	*Pyrococcus furiosus*	100	17.5	15.7	0.41

**Figure 1:**
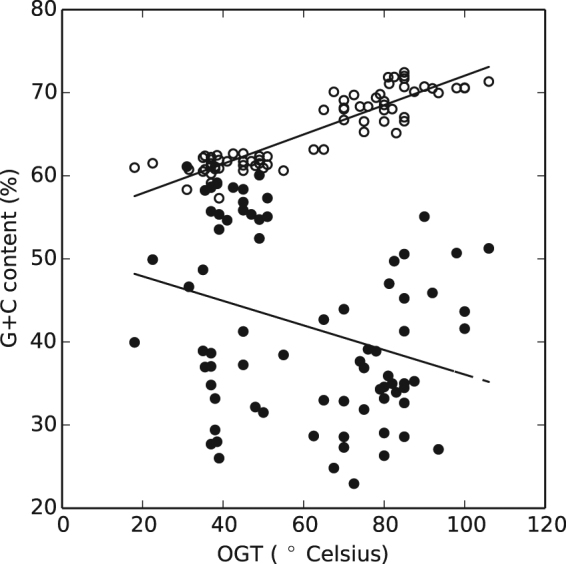
G + C content for tRNA coding and promoter regions. G + C content was analyzed either in the region coding for the tRNA itself (empty circles) or in a promoter region of the same length for each of the genomes in our dataset (filled circles). Coding regions show a clear increase with temperature (R = 0.91 p = 4.9 × 10^−30^), while promoters region do not (R = −0.3 p = 7 × 10^−3^).

We then decided to compare regions of similar length and location in the genomes regarding the trends in GC composition. We choose to compare tRNAs genes (circa 80 bp) with their corresponding promoter region of exactly the same length. Results are presented in Fig. 1 where a clear correlation is observed for the coding region while no correlation (beyond the apparent negative trend) is observed for the promoter.

Optimal growth temperature has been informed to be inversely correlated with genome size^14^. Our data is consistent with that report and is presented in Figure S2, were a disperse but clearly negative trend is observed. It is also expected that genome size correlates with the number of ORFs. Thus, we compared genomes size with number of ORFs obtaining a graph that fits to a linear correlation with *R* = 0.968, Figure S2, inset. Lastly, it has been reported that information content in a binding site is about what is needed to locate that site in its genome^15^. Combining the above considerations it could be expected that organisms living at higher temperature, have smaller genomes, with fewer ORFs, requiring less IC for proteins to bind its sites. However, since less ORFs means less possible target sites for TBP, the ratio of sites to genome size is constant, therefore the amount of information needed to find one of the sites in the genome remains constant. Unexpectedly, it is not what we observe in our dataset, where IC clearly increases with optimal growth temperature (see Optimal Growth Temperature section).

### Finding Motifs

**Figure 2:**
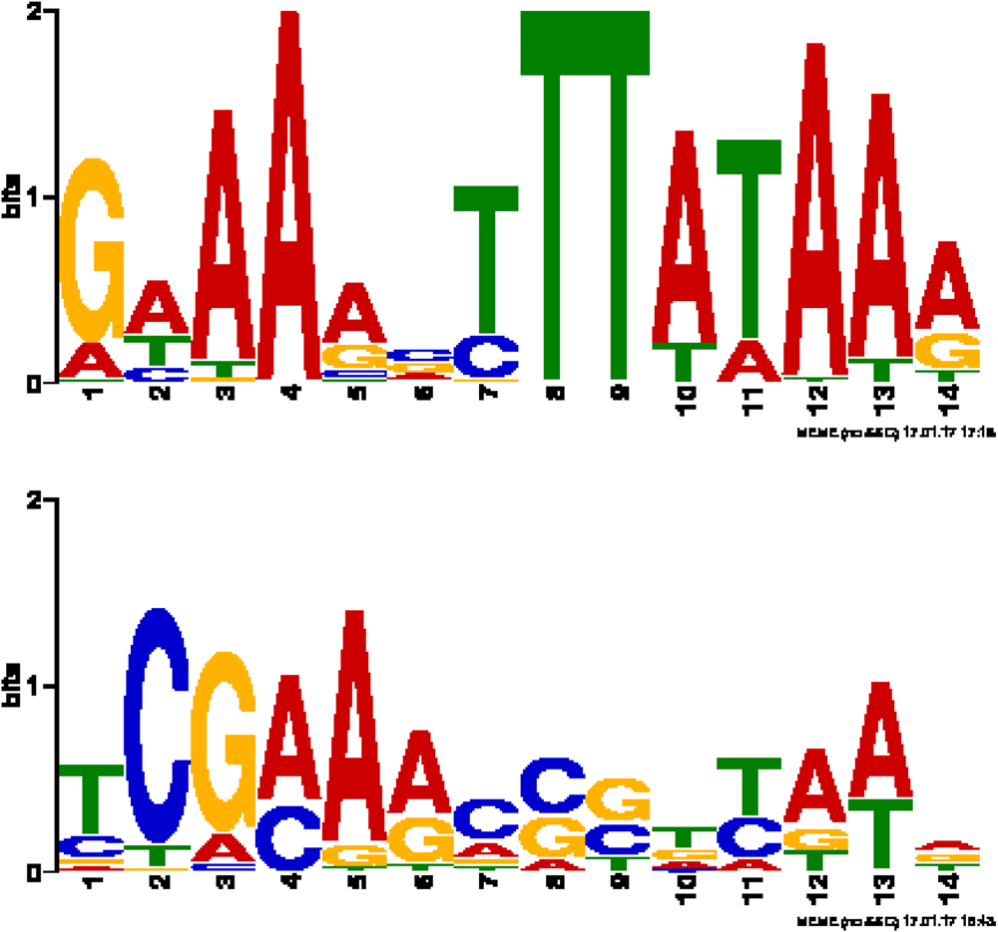
Best and worst TATA’s logos. Lowest (top) and highest (bottom) e-score motif of the TBP binding site (TBS) for 39 archaeal genomes. TBS are represented as sequence logos. 100 bp upstream regions of tRNAs were collected for each genome and were analyzed by MEME to identify TBS. For a full list of obtained logos refer to Figure S12.

TBP binding motifs were elicited for each genome by performing motif discovery with MEME on the upstream regions of tRNA genes (see methods). To avoid false positives (similar sequences that do not correspond to TBS) we narrowed our search by distance to the transcription start site. We considered 100 bp upstream of the ORF which is sufficient to include 95% of the sites, as we show on Figure S3, the modal distance is 40 bp, consistent with the reported distance for TATA box^16^. This criterion includes most sites and reduces the noise produced by sequences that might include motifs somewhat similar to a TATA box, but that would not be functional as such. Using 500 bp cut-off produces in similar results, as we show on Figure S4. Resulting TBP binding sites for each species are presented as sequence logos in Fig. 2 and Figure S12).

We characterized the core promoter region for different archaeal species in terms of regular expression, base composition and information content (IC) (Table 1). We confirmed that the motifs are short and not part of a larger conserved unit, by visualizing the conservation of the aligned sequences upstream and downstream of the shown motifs (Figure S12). This suggest that TBS length is rather invariable, at least, within the range of the studied temperatures.

Highest scoring motifs for each species vary between motifs easy to be identified as TATA boxes (ie: consensus sequence contains TATA) and hardly recognizable ones (Fig. 2 for examples and Figure S12 for a full set of promoter’s logos). Notably, there are some taxa exhibiting TBP protein but lacking a TATA motif ^6^. Those cases were excluded from our analysis to avoid comparing IC between binding sites that may be recognized by other proteins and not TBP. We observed that some of the motifs include a sequence [GC][GC][GA]CGCC, which is also present in the methanogen archaea. This motif is consistent with the BRE site, located upstream of the TATA box and crucial in transcription initiation. It is not surprising that motifs of sites with a lowly conserved TATA box looks like biased towards a BRE site.

We tested whether some positions of the motif were more conserved than others, suggesting higher importance in protein-DNA interaction of those positions, possibly related to adaptation to higher temperatures (Table S2).

We performed a clustering based on motif divergency and show that they do not overlap with either temperature clusters or with phylogeny clades in Figures S9 and S10.

### Optimal Growth Temperature

**Figure 3:**
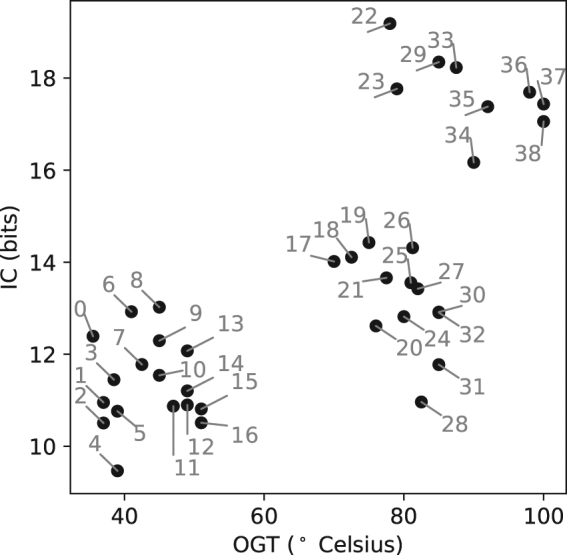
Information content correlates with optimal growth temperature. Information content of the predicted TBS motif on 100bp window, for 39 archaeal genomes is plotted against optimal growth temperature. Each dot represents one species average. Numbering for each dot refers to the species/motifs in Table 1

Temperatures at which biochemical processes occur may affect reaction rates and molecule conformations among other factors. Thus, the range of extreme temperatures at which an organism is able to live could tell us something about underlying processes. Even though maximal growth temperature could have been more informative, information on this parameter is scarce. We found a positive correlation between optimal growth temperature and information content as shown in Fig. 3. This correlation is significant (*R* = 0.67, *p* = 3.5 × 10^−6^), but with no obvious explanation for its source. Although we cannot attribute this trend to a particular process nor adjust it to a linear or sigmoidal function, it is evident that there is a trend to increase IC with temperature with a change around 75 degrees where a much higher slope is seen in Fig. 3.

In Figure S1 we showed that G + C composition does not increase with OGT. Combining that result with the observation that information content increases with OGT (Fig. 3), it is expected that IC may decrease at higher genomic G + C content, which is shown in Figure S5, where a negative slope is seen.

We found that the information content of the binding site for the TATA binding protein is higher at higher temperatures for the thermophile Archaea analyzed and suggest that it could be the same for further species, yet to be characterized. It remains to be proved whether this property is also valid for other DNA binding proteins and/or for TBP-TBS in other kingdoms of life. Specific protein-DNA interactions may involve direct contacts between aminoacids with bases and indirect readouts through conformational effects, either from the DNA^17^ or the protein side^18^. TBP recognizes its target sites mostly by indirectly reading the DNA^19^ and thus may be more affected by temperature than proteins relying mostly or exclusively on direct reading.

Rsequence is defined as the amount of information contained in a set of aligned sequences and is approximately equal to Rfrequency, which is the minimum information needed to find a sequence on a genome^15^. That means, according to Schneider’s 1986 article^15^, that the information content (Eq. 1) of a binding site is just about the minimum information needed to find it on a genome. This appears to be true for most studied prokaryotic cases. We propose there may be a selective pressure towards a small limited set of sequences with higher binding affinity.

To determine Rfrequency’s (View Eq. 1) variation with genome size, we considered as *γ* the number of ORFs and as *ω* the genome size. Since in our dataset *γ* is approximately a linear function of *ω* (Figure S2), by applying equation 1 it is possible to estimate Rfrequency:

$$Rfreq\approx 10\,bit/site$$

If we further assume the reported 70% efficiency for molecular machines^20^, applies to Archaea TATA sites, the energy of binding for these 10 bit sites would be about 10/0.7 = 14 bits per site or a *K*_*D*_ of 2^14^ = 16384. Computing the actual efficiency will require measuring the *K*_*D*_ of the sites.

Values above 15 bits at higher temperatures suggest that some assumptions are not valid above 75–80° or, more interesting, that a yet unidentified process (maybe thermal noise) interferes with TBP’s binding site location at these temperatures. It would be interesting to inquire whether this interference could uncover a biological mechanism to stabilize DNA at very high living temperatures.

## Conclusion

The temperature at which an organism lives, affects many biochemical processes. It is often mentioned in lectures that G + C content increases with temperature. We show that this correlation is present in functional RNAs but not at a whole genome level. Particularly, we showed that this correlation is present in our set of tRNAs and absent in its corresponding promoter regions. Other correlations as number of ORFs proportional to genome size or the tendency to reduced genome sizes at higher temperatures are also consistent with our results. Interestingly our analysis shows that information content notably increases at higher OGT, although no explanation for this fact is available. Information content is a parameter similar in this context to Rsequence, that indicates a selective pressure acting upon the site by means of a recognizer^15^. According to our estimation based on the number of sites and size of the genome Rfreq(see methods), approximately equals to the IC for low temperature archaea. The ratio between genome length and the number of binding sites for a recognizer could potentially affect IC required for a binding site. However, it has been observed that genome size is proportional to the number of ORFs. Thus, the information needed to find a TBP site is approximately the same, independently of genome size. As we have shown the value of Rseq varies in our dataset, colliding with the classical proposition of Rfreq = Rseq, from molecular information theory^21^.

On one hand, it is reasonable to assume that information content in DNA sequences is subject to selective pressure, because under neutrality it would be lost due to mutation^15^. On the other hand, it has been described that a binding site contains only enough information to be found in its genome^15^. Notably, we show that the information content is much higher than what is needed for a recognizer to find it on a genome considering only its size. Thus, there must be another source for information’s conservation. It could be evolutionary, energetic or a combination of both. It has been reported that organisms living in extreme temperatures have a strong tendency to slower divergence^22^. This would produce less binding site divergence and, thus, higher IC. In this scenario the higher IC might be a consequence of a higher temperature, an environmental factor that imposes a constraint on the number of possible functional sites. It is also envisioned that at high temperatures DNA binding discrimination between specific and nonspecific sites may be diminished. Thus, requiring more IC to bind real targets. Another issue to be considered is that at temperatures close to water boiling point organisms may have develop anti-boiling systems as analogy to anti-freeze systems existing in psychrophiles living at temperatures below ice formation. We suggest to limit the application of the established relation between Rsequence and Rfrequency from molecular information theory^15^ to a range of temperatures, or to extend the theory to take into account the effect of temperature if the observed trend was intrinsic to molecular recognition.

## Materials and Methods

### Genomes

All genomes where retrieved from NCBI genomes database. Genomes used for this analysis comprise the 78 archaeal genomes of nucleotide core, that where at least partially annotated, completed, and published in the database by December 26th, 2016. Sequences below 1 Mbp were discarded. All genomes had at least ORF annotation. Sequences from candidate species that have not been accepted yet were excluded. Although we included methanoarchaea taxons in Table S1, they were excluded from further analysis, since there are no TBS motif reported in those groups^6^.

### Promoter sequence sets

Transfer RNA (tRNA) were retrieved from annotated genomes, available in GenBank format from NCBI. In order to obtain a balance between a short upstream sequence and a long enough promoter region to be analysed by MEME, the 100 bp upstream region from each tRNA was retrieved. Genomes exhibit an average of 45 tRNA independently of their OGT, Figure S6. Motifs instances are available as a supplementary file.

### Motif discovery

Motif discovery was performed on the collection of 100 bp upstream regions of each genome. MEME^23^ software was used with the following parameters: *-dna -nmotifs 10 -nsites X -w 14 -maxsize 10000000*. X is the number of sequences used, meaning there must be at least one occurrence of the motif per sequence. *“-w”* is used because we expect from previous model that the length of a TBS is 14 bp^6^. Best scoring motif was selected for each species fulfilling the following requirements: (a) Had an e-value threshold of 1 × 10^−6^, (b) Was present in at least 50% of the sequences, and (c) Had a G + C content lower than 50%. Even a threshold of 1 × 10^−10^ would not change the results, as seen in Figure S7. The information content of the motifs was calculated as:

$$IC=\sum _{b=A}^{T}\sum _{l=0}^{L}f(b,l)\mathrm{log}\,\frac{f(b,l)}{P(b)}$$

Where f(b, l) is the frequency of the base *b* at position *l*. P(b) was either 0.25 for IC, or the relative frequency of that base on the genome for RE. We inform IC in Fig. 3 as it is the most widely used metric. In Figure S11 we also inform the more accurate metric Rseq, that considers small sample size.

### Optimal growth temperature data compilation

Optimal Growth Temperature was considered as the published OGT. When it was not available (twelve species) it was approximated as the average between published maximal and minimal growth temperatures. When we found more than one source of information, the average of the multiple sources was used. When the only available information was vague, describing a strain as mesophilic, we excluded this strain from further analysis. Since generally there was no information about each strain, information available for the same species was used.

### Rfrequency estimation

Rfrequency is the amount of information needed to find a set of binding sites out of all the possible sites in the genome. Rfrequency only depends on genome size, and the number of sites in the genome. By definition Rfrequency is^15^:

1 $$\begin{array}{rr}Rfreq & =-{\mathrm{log}}_{2}\frac{\gamma }{\omega }\\ When:\gamma \approx K\omega \to Rfreq(\omega ) & \approx -{\mathrm{log}}_{2}K\end{array}$$

*γ* is the number of sites and, *ω* is the number of bases in the genome.

For each genome in our dataset, *γ* and *ω* were plotted, to approximate $\frac{\gamma }{\omega }$ by a linear regression (Figure S2).

## Electronic supplementary material

Supplementary information, figures and text

Dataset 1
