# Supplementary material for: Core promoter information content correlates with optimal growth temperature
Source: Sci Rep. 2018 Jan 22;8:1313. doi: 10.1038/s41598-018-19495-8 (PMC5777992; doi:10.1038/s41598-018-19495-8)
Supplement: Supplementary file 1 — Supplementary information, figures and text [file 41598_2018_19495_MOESM1_ESM.pdf]

Supplementary material for ”Core promoter information content correlates with optimal growth temperature”

Ariel A. Aptekmann[a] **and Alejandro D. Nadra**[a,b1]

[a]Universidad de Buenos Aires. Facultad de Ciencias Exactas y Naturales. Departamento de Química Biológica, Laboratorio de Bioquímica Estructural. Buenos Aires, Argentina.
CONICET. Instituto de Química Biológica de la Facultad de Ciencias Exactas y Naturales (IQUIBICEN). Intendente Güiraldes 2160, C1428EGA, Buenos Aires, Argentina.
[b]Universidad de Buenos Aires. Facultad de Ciencias Exactas y Naturales. Departamento de Fisiología, Biología Molecular y Celular , Laboratorio de Bioquímica Estructural. Buenos Aires, Argentina.

ABSTRACT

The subtle mechanisms by which protein-DNA interactions remain functional across a wide range of temperatures are largely unknown. In this work, we manually curated available information relating fully sequenced archaeal genomes with organism growth temperatures. We built a motif that represents the core promoter of each species and calculated its information content. We then studied the relation between optimal growth temperature (OGT) and information content (IC) in the promoter region.We found a positive correlation between G+C content and OGT in tRNA regions and not in overall genome. Furthermore, we found that there is a positive correlation between information content and optimal growth temperatures in Archaea. This can't be explained by an increased C+G composition nor by other obvious mechanisms. These findings suggest that increased information content could produce a positive fitness in organisms living at high temperatures. We suggest that molecular information theory may need to be adapted for hyperthermophiles.

Supplementary Material

We originally considered for this study all NCBI's reference archaeal genomes. We later on excluded Methane generating Archaea, as there are some doubts about the mechanism of transcription initiation in these groups[1]. The taxa, that have a TBP, but are excluded for this reason are the following: NC 000909, NC 014222, NC 014253, NC 014002, NC 017034, NC 014658, NC 009515 , NC 015416 , NC 013665 , NC 009464 , NC 014122 , NC 003901 , NC 014408 , NC 009051 , NC 015636 , NC 015562 , NC 017527 , NC 007355 , NC 013790 , NC 013407 , NC 018876 ,NC 005791.

We show that the G+C content of 16s rRNA but not whole genome sequences correlates with OGT in Figure S 1. This might help to explain why the usual perception states that G+C content correltates with OGT.

We show that OGT deacreses with increasing genome size in Figure S 2 and that the number of ORFs correlates with genome size on the inset in the same Figure S 2.

We show that most of the sites are within 100 bp shown in Figure S 3 and that running a similar analysis to the one performed in Fig 3 but using only 500 bp yields similar results in Figure S 4.

We show in Figure S 5 that in the region surrounding the transcription start sites (TSS) a negative correlation between G+C % and IC is visible.

We also include a figure about the number of tRNA in the different genomes considered in Figure S 6 and show it is quite evenly distributed along the optimal temperature range.

We show in Figure S 7 that using a more restrictive threshold for the score of the motifs, does not change the tendency.

As most of our results derive on putative motifs identified by MEME and to avoid biases produced by that tool, we have obtained putative binding sequences in a MEME independent way. We have directly recovered and aligned the regions between 50 to 20 bp upstream of TSS of each tRNA for the 39 Archaea genomes included in Fig 3. For the alignment we used Clustalw under the parameters -type=DNA -pwgapopen=500, and then calculated Rseq applying small sample correction (using exact method[2]). As seen on figure S8, the source of the motifs does not change the observed tendency in Figure 3.

We then analysed if the motif divergency clusters maps neatly onto a phylogenic tree, shown in Figure S 9 or if they are related to the temperature clusters shown in Figure S 10. Although not an exhaustive analysis, at least it shows that the motifs found are not grouped by temperature range or phylogenetic groups.

We show that applying small sample correction (using exact method[2]) does not change the observed tendency, in Figure S 11.

We include motifs instances as supplementary .fasta file, where each sequence name includes the NC code, version, starting position, and orientation of the sequence.

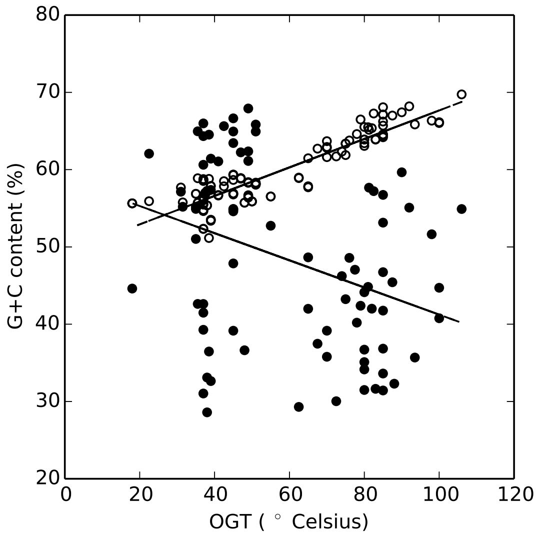

Supplementary Figure 1. 16S rRNA but not full genome composition increases its G+C content as a function of temperature. All archaeal genomes in our dataset were analyzed in terms of base compositions considering full genome (filled circles) or 16S rRNA (empty circles) and plotted against OGT. No correlation was found for full genome composition obtaining (R $=-0.21$, p $=2\times10^{-4}$) while a clear correlation was found for 16S rRNA (R =0.91, p $=9.8\times10^{-49}$).

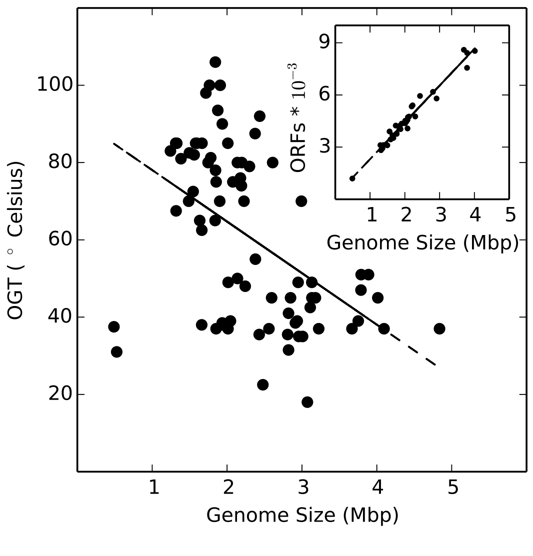

Supplementary Figure 2. Genome size (Eq.1 ω) and number of ORFs (Eq.1 γ) decrease at higher temperatures. Optimal growth temperature for our set of organisms was compared to its corresponding genome's size presenting a negative slope ($R=-0.5$, $p=4.6\times10^{-6}$). Inset: The amount of ORFs were correlated with genome size ($R=0.968$, $p=4.2\times10^{-27}$).

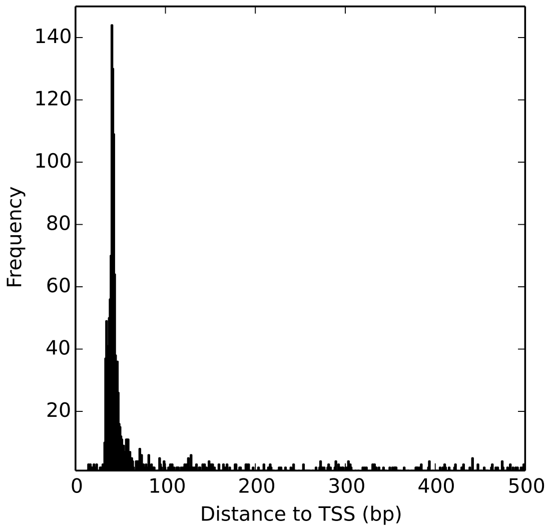

Supplementary Figure 3. Histogram of the distances from each of the TATA motif to the beginning of its corresponding TSS. Combined data from all the species is shown. Mode is -40 bp, consistent with what was expected for core promoter region.

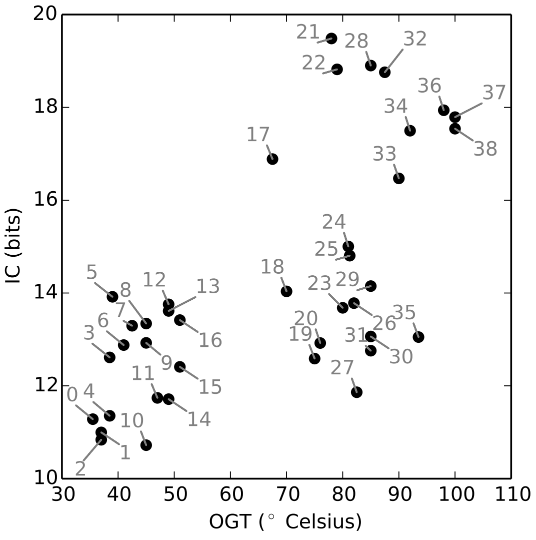

Supplementary Figure 4. Information content correlates with optimal growth temperature. Information content of the TBS for 39 archaeal genomes is plotted against optimal growth temperature. Each dot represents one species average IC calculated upon the motifs obtained with a 500 bp window instead of a 100 bp window as is shown in the main text.

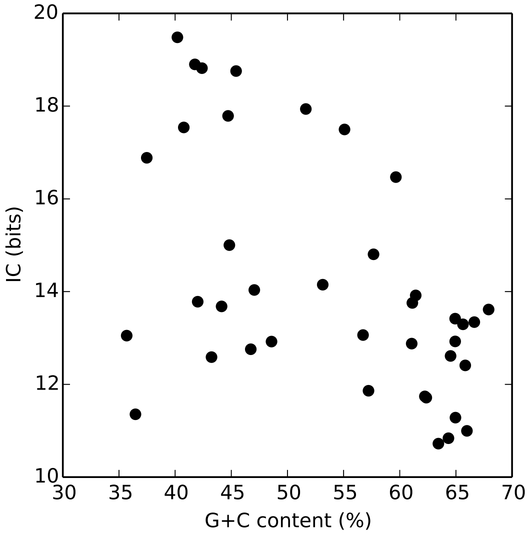

Supplementary Figure 5. Information content does not increase with genome G+C content. G+C % and information content were calculated for each genome and plotted. Variance in information content seems to be negatively correlated to G+C composition, with a correlation coefficient of -0.67 and a p-value of 7×10^{-5}

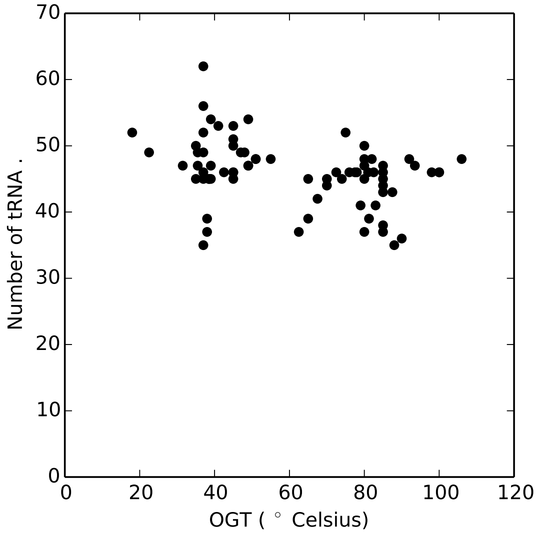

Supplementary Figure 6. For all Archeal genomes the number of tRNA for an organism, is relatively uniform with varying OGT in our dataset. This accounts only for annotated tRNA.

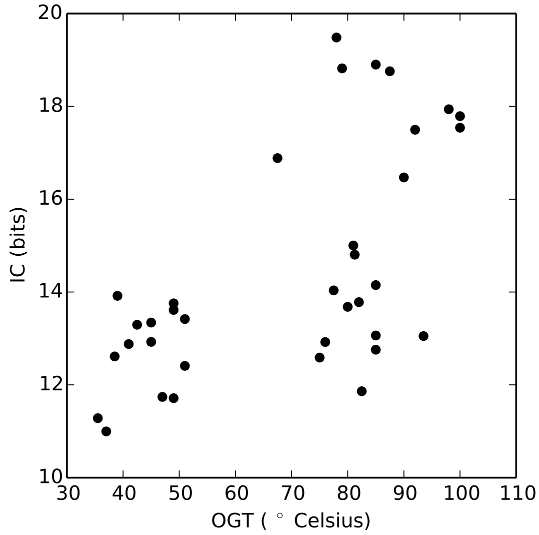

Supplementary Figure 7. Information content correlates with optimal growth temperature. Information content of the TBS for 36 archaeal genomes is plotted against optimal growth temperature. We changed the e-value threshold to 10^{-10}. In the main text we perform a similar analysis with an e-value cutoff of 10^{-3}), being more restrictive does not change the trend in the results.(But excludes 3 genomes and therefore the number of datapoints is 36 not 39)

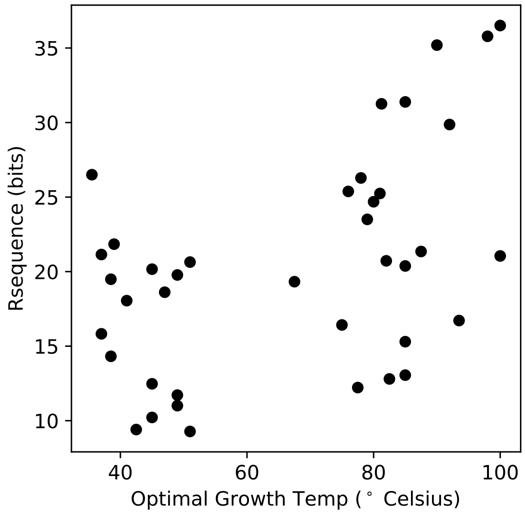

Supplementary Figure 8. Rsequence vs OGT of the regions between 50 to 20 upstream of TSS of each tRNA for each of the 39 Archaea genomes included in Fig 3. We aligned sequences using Clustalw under the parameters -type=DNA -pwgapopen=500, and then calculate Rseq applying small sample correction (using exact method[2]). Correlation coefficient ($R = 0.5 p = 0.001$).

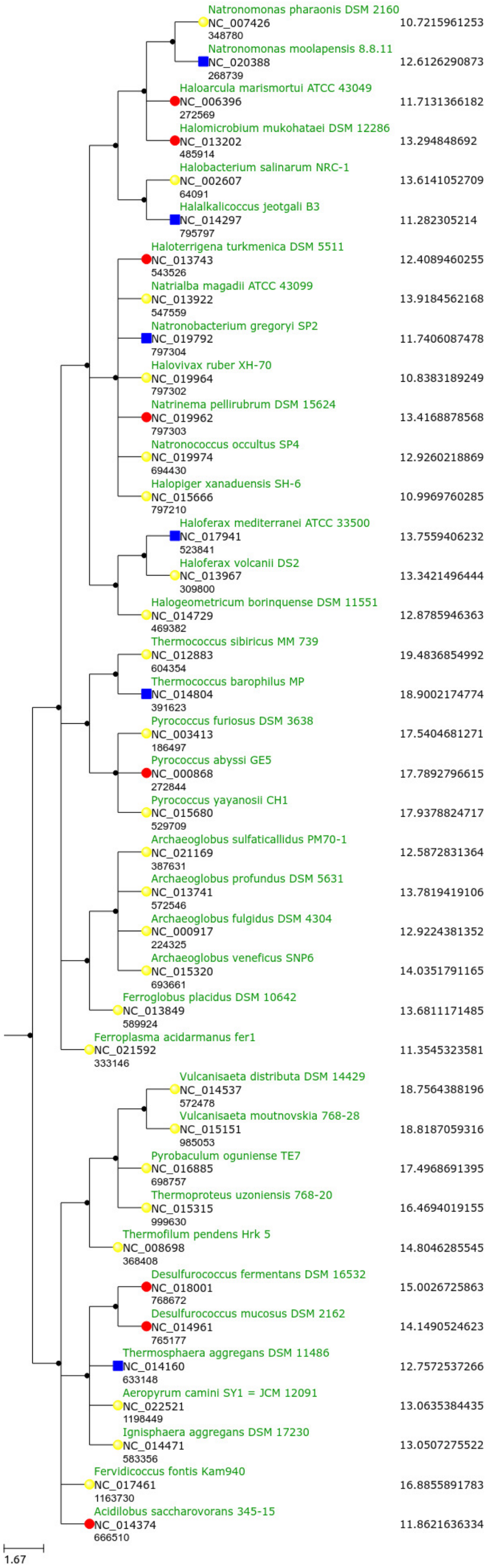

Supplementary Figure 9. NCBI's phylogenetic tree of the 39 Archea genomes included in Fig 3. The symbols on the left of each NC code denote they belong to a certain cluster. Clusters where generated using a k-medoids algorithm with n=3, using as distance between motifs TOM-TOM e-values.

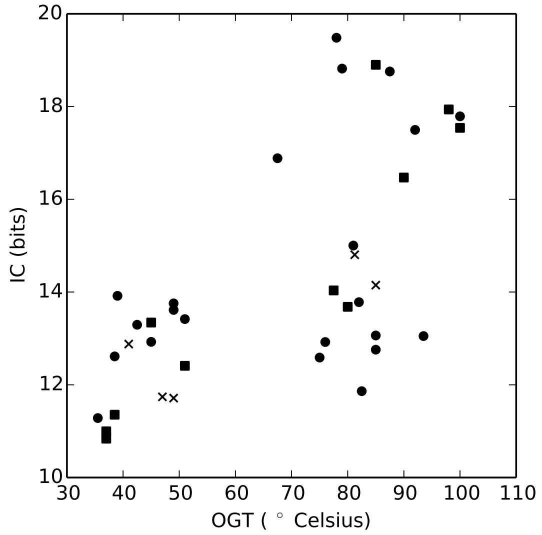

Supplementary Figure 10. Information content plotted against optimal growth temperature for the 39 archeal genomes in Fig 3, but each symbol (square, circle, cross) represents a cluster formed with the same clustering method as in Figure S 9.

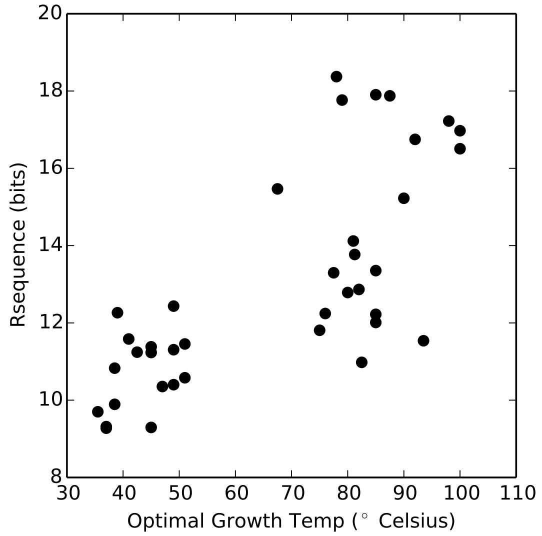

Supplementary Figure 11. Rsequence correlates with optimal growth temperature. We calculated Rseq considering small sample correction.[2].

Supplementary Table 1. Optimal Growth Temperature for all reference completed archaeal genomes.

Species	Opt. Growth Temp (°C)
Acidianus hospitalis	80[3]
Acidilobus saccharovorans	82[4]
Aciduliprofundum boonei	70[5]
Aeropyrum camini	85[6]
Archaeoglobus fulgidus	76[7]
Archaeoglobus profundus	82[8]
Archaeoglobus sulfaticallidus	75[9]
Archaeoglobus veneficus	77[10]
Caldisphaera lagunensis	72[11]
Caldivirga maquilingensis	85[12]
Cenarchaeum symbiosum	10[13]
Desulfurococcus fermentans	81[14]
Desulfurococcus mucosus	85[15]
Ferroglobus placidus	80[16]
Ferroplasma acidarmanus	38[17]
Fervidicoccus fontis	67[18]
Halalkalicoccus jeotgali	35[19]
Haloarcula hispanica	37[20]
Haloarcula marismortui	49[21]
Halobacterium salinarum	49[21]
Haloferax mediterranei	49[21]
Haloferax volcanii	45[21]
Halogeometricum borinquense	41[21]
Halomicrobium mukohataei	42[22]
Halopiger xanaduensis	37[23]
Haloquadratum walsbyi	45[24]
Halorubrum lacusprofundi	34[25]
Haloterrigena turkmenica	51[21]
Halovivax ruber	37[26]
Ignicoccus hospitalis	86[27]
Ignisphaera aggregans	93[28]
Metallosphaera cuprina	65[29]
Metallosphaera sedula	74[30]
Methanobrevibacter ruminantium	39[31]
Methanobrevibacter smithii	37[32]
Methanocaldococcus infernus	85[33]
Methanocaldococcus jannaschii	85[34]
Methanocaldococcus vulcanius	80[35]
Methanocella arvoryzae	45[36]
Methanocella conradii	55[37]
Methanocella paludicola	35[38]
Methanococcus maripaludis	38[39]
Methanococcus voltae	38[40]
Methanoculleus marisnigri	22[41]
Methanohalobium evestigatum	48[42]
Methanohalophilus mahii	37[43]
Methanolobus psychrophilus	18[44]
Methanomethylovorans hollandica	35[45]
Methanoregula formicica	31[46]
Methanosaeta concilii	35[47]
Methanosaeta harundinacea	37[48]
Methanosalsum zhilinae	45[49]
Methanosarcina barkeri	37[50]
Methanosarcina mazei	37[51]

Isolated from cow rumen, at 40 °C[31]

Supplementary Table 1. Continuation.

Species	Opt. Growth Temp (°C)
Methanothermobacter marburgensis	65[52]
Methanothermococcus okinawensis	62[53]
Methanothermus fervidus	83[54]
Methanotorris igneus	88[55]
Natrialba magadii	39[42]
Natrinema pellirubrum	51[21]
Natronobacterium gregoryi	37[42]
Natronococcus occultus	45[21]
Natronomonas moolapensis	38[56]
Natronomonas pharaonis	45[21]
Pyrobaculum aerophilum	100[57]
Pyrobaculum arsenaticum	84[58]
Pyrobaculum oguniense	92[59]
Pyrococcus abyssi	96[60]
Pyrococcus furiosus	100[61]
Pyrococcus yayanosii	98[62]
Pyrolobus fumarii	106[63]
Staphylothermus hellenicus	85[64]
* *Sulfolobus acidocaldarius*	80[65]
Sulfolobus islandicus	80[66]
Sulfolobus solfataricus	80[67]
Thermococcus barophilus	85[68]
Thermococcus gammatolerans	88[69]
Thermococcus kodakarensis	95[70]
Thermococcus onnurineus	80[71]
Thermococcus sibiricus	78[72]
Thermofilum pendens	87[73]
Thermogladius cellulolyticus	84[74]
Thermoplasmatales archaeon	40 *[31]
Thermoproteus uzoniensis	90[75]
Thermosphaera aggregans	85[76]
Vulcanisaeta distributa	87[77]
Vulcanisaeta moutnovskia	79[78]

* Isolated from cow rumen, at 40 °C[31]

Supplementary Table 2. Average information content by position, it shows that some positions might be more relevant than others. For each position the Pearson's correlation coefficient of IC vs OGT, and its two tailed p-value are shown.

Position	Average IC	Pearson R	P value
1	1	-0.187	0.254
2	1.15	-0.421	0.00762
3	1.01	-0.0835	0.613
4	1.15	0.367	0.0214
5	1.07	0.296	0.0677
6	0.786	-0.0718	0.664
7	0.934	-0.16	0.329
8	1.16	0.157	0.341
9	1.26	0.677	**2.17e-06**
10	1.08	0.395	0.0127
11	1.12	0.254	0.119
12	1.02	0.262	0.107
13	1.13	0.0185	0.911
14	1.07	-0.139	0.399

IC: Average IC for all motifs; R: Pearson's correlation value between IC and OGT; P: two tailed p-value calculated for each position. Positions are relative to the logo shown on Fig 2

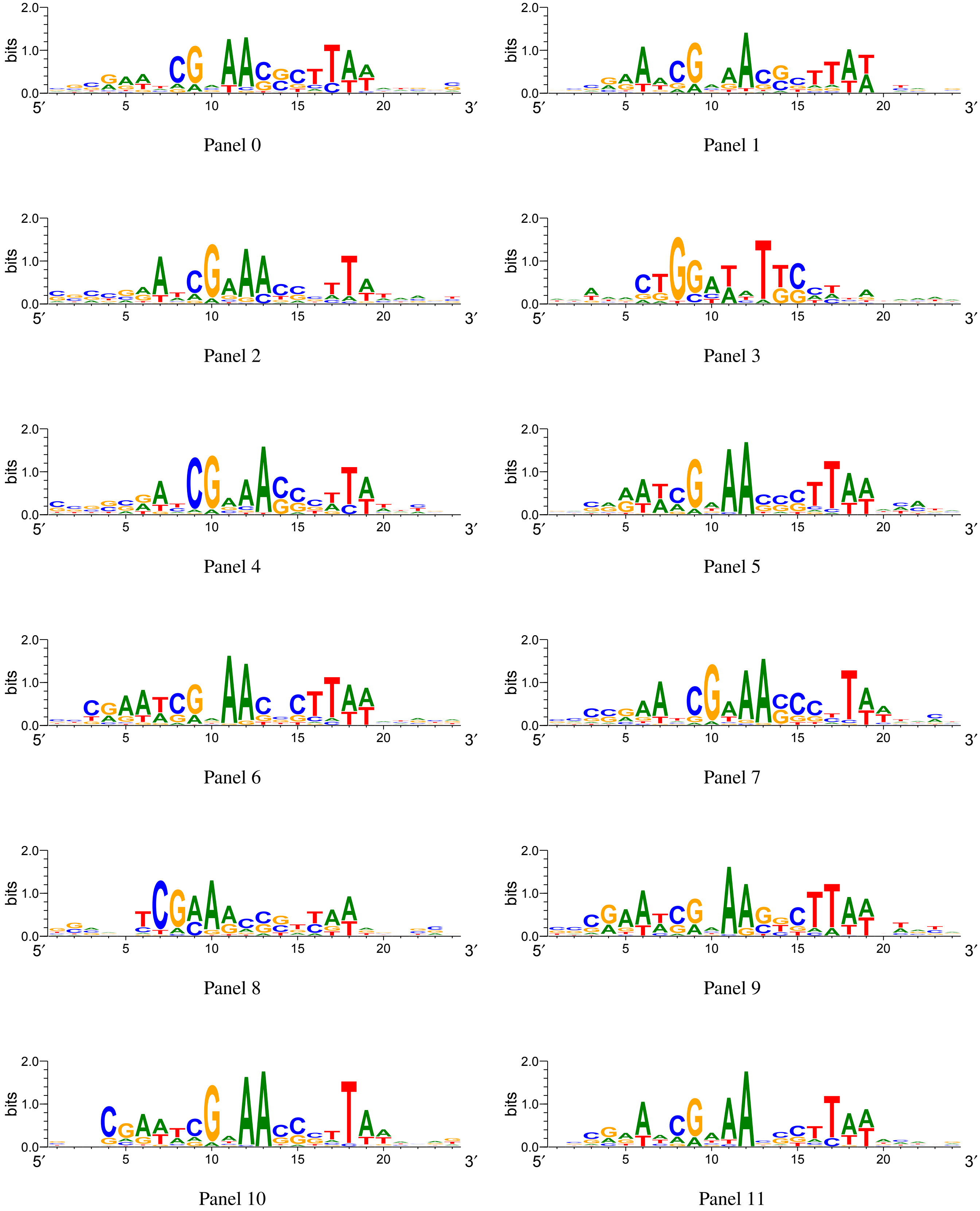

Supplementary Figure 12. Core promoter Motifs for all studied archaea. Numbering corresponds to Figure 3 and Table 1 .

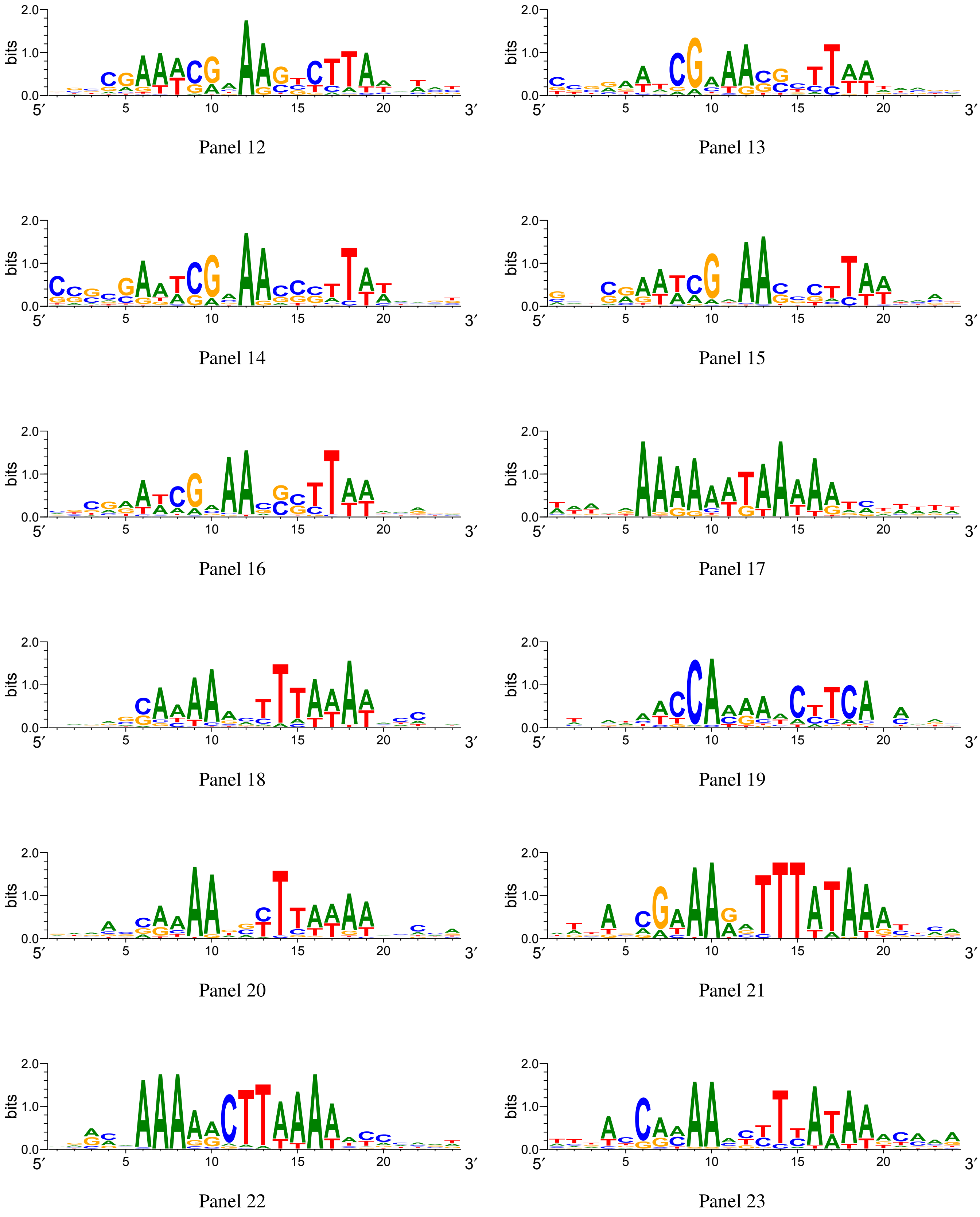

Supplementary Figure 12. Core promoter Motifs for all studied archaea. Numbering corresponds to Figure 3 and Table 1 .

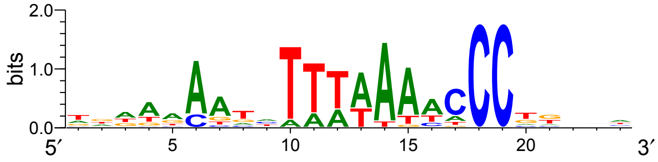

Panel 24

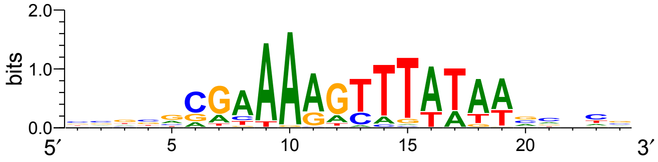

Panel 25

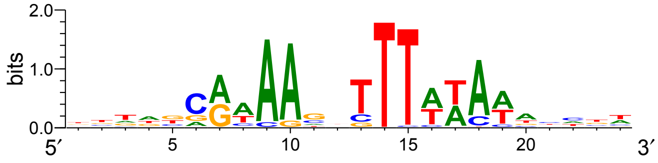

Panel 26

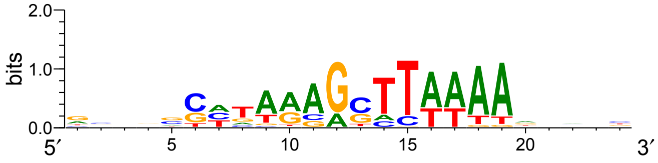

Panel 27

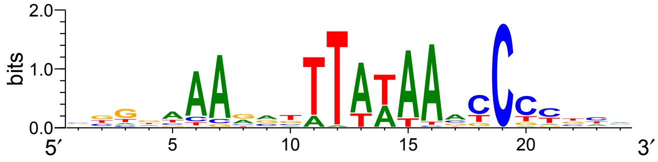

Panel 28

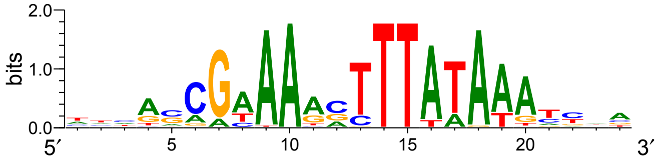

Panel 29

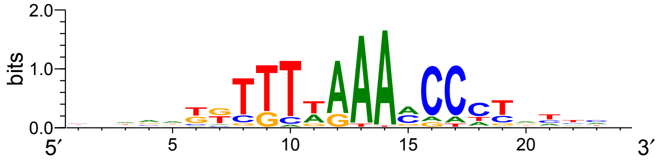

Panel 30

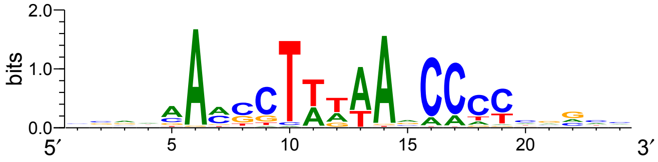

Panel 31

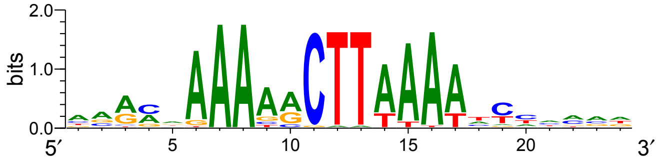

Panel 32

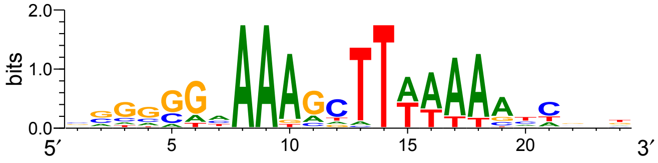

Panel 33

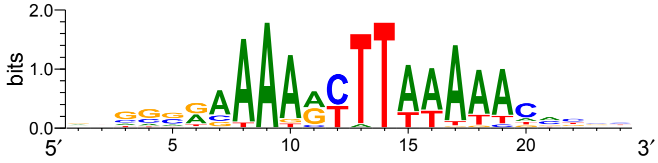

Panel 34

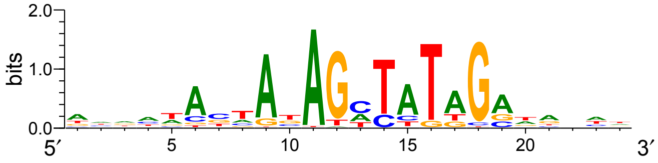

Panel 35

Supplementary Figure 12. Core promoter Motifs for all studied archaea. Numbering corresponds to Figure 3 and Table 1 .

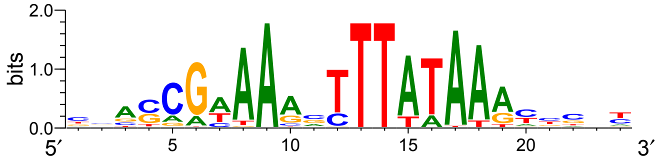

Panel 36

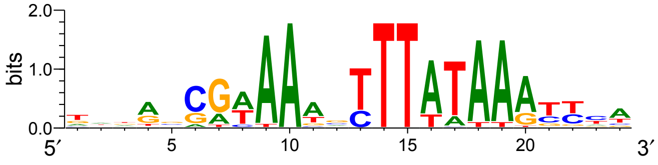

Panel 37

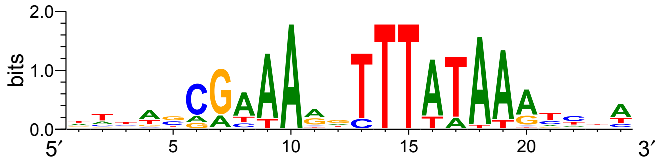

Panel 38

Supplementary Figure 12. Core promoter Motifs for all studied archaea. Numbering corresponds to Figure 3 and Table 1 .

References

1. Blum, P. *Archaea: ancient microbes, extreme environments, and the origin of life*, vol. 50 (Gulf Professional Publishing, 2001).
2. Schneider, T. D., Stormo, G. D., Gold, L. & Ehrenfeucht, A. Information content of binding sites on nucleotide sequences. *J. molecular biology* **188**, 415–431 (1986).
3. You, X.-Y. *et al.* Genomic analysis of acidianus hospitalis w1 a host for studying crenarchaeal virus and plasmid life cycles. *Extrem.* **15**, 487–497 (2011).
4. Prokofeva, M. I. *et al.* Isolation of the anaerobic thermoacidophilic crenarchaeote acidilobus saccharovorans sp. nov. and proposal of acidilobales ord. nov., including acidilobaceae fam. nov. and caldisphaeraceae fam. nov. *Int. journal systematic evolutionary microbiology* **59**, 3116–3122 (2009).
5. Perner, M., Gonnella, G., Kurtz, S. & LaRoche, J. Handling temperature bursts reaching 464 c: different microbial strategies in the sisters peak hydrothermal chimney. *Appl. environmental microbiology* **80**, 4585–4598 (2014).
6. Nakagawa, S., Takai, K., Horikoshi, K. & Sako, Y. Aeropyrum camini sp. nov., a strictly aerobic, hyperthermophilic archaeon from a deep-sea hydrothermal vent chimney. *Int. journal systematic evolutionary microbiology* **54**, 329–335 (2004).
7. Beeder, J., Nilsen, R. K., Rosnes, J. T., Torsvik, T. & Lien, T. Archaeoglobus fulgidus isolated from hot north sea oil field waters. *Appl. environmental microbiology* **60**, 1227–1231 (1994).
8. Burggraf, S., Jannasch, H. W., Nicolaus, B. & Stetter, K. O. Archaeoglobus profundus sp. nov., represents a new species within the sulfate-reducing archaebacteria. *Syst. Appl. Microbiol.* **13**, 24–28 (1990).
9. Steinsbu, B. O. *et al.* Archaeoglobus sulfaticallidus sp. nov., a thermophilic and facultatively lithoautotrophic sulfate-reducer isolated from black rust exposed to hot ridge flank crustal fluids. *Int. journal systematic evolutionary microbiology* **60**, 2745–2752 (2010).
10. Huber, H., Jannasch, H., Rachel, R., Fuchs, T. & Stetter, K. O. Archaeoglobus veneficus sp. nov., a novel facultative chemolithoautotrophic hyperthermophilic sulfite reducer, isolated from abyssal black smokers. *Syst. Appl. Microbiol.* **20**, 374–380 (1997).
11. Itoh, T., Suzuki, K., Sanchez, P. & Nakase, T. Caldisphaera lagunensis gen. nov., sp. nov., a novel thermoacidophilic crenarchaeote isolated from a hot spring at mt maquiling, philippines. *Int. journal systematic evolutionary microbiology* **53**, 1149–1154 (2003).
12. Itoh, T., Suzuki, K.-i., Sanchez, P. C. & Nakase, T. Caldivirga maquilingensis gen. nov., sp. nov., a new genus of rod-shaped crenarchaeote isolated from a hot spring in the philippines. *Int. J. Syst. Evol. Microbiol.* **49**, 1157–1163 (1999).
13. Preston, C. M., Wu, K. Y., Molinski, T. F. & DeLong, E. F. A psychrophilic crenarchaeon inhabits a marine sponge: Cenarchaeum symbiosum gen. nov., sp. nov. *Proc. Natl. Acad. Sci.* **93**, 6241–6246 (1996).
14. Susanti, D. *et al.* Complete genome sequence of desulfurococcus fermentans, a hyperthermophilic cellulolytic crenarchaeon isolated from a freshwater hot spring in kamchatka, russia. *J. bacteriology* **194**, 5703–5704 (2012).
15. Zillig, W. *et al.* Desulfurococcaceae, the second family of the extremely thermophilic, anaerobic, sulfur-respiring thermoproteales. *Zentralblatt für Bakteriologie Mikrobiologie und Hyg. I. Abt. Orig. C: Allgemeine, angewandte und ökologische Mikrobiologie* **3**, 304–317 (1982).
16. Hafenbradl, D. *et al.* Ferroglobus placidus gen. nov., sp. nov., a novel hyperthermophilic archaeum that oxidizes fe2+ at neutral ph under anoxic conditions. *Arch. Microbiol.* **166**, 308–314 (1996).
17. Dopson, M., Baker-Austin, C., Hind, A., Bowman, J. P. & Bond, P. L. Characterization of ferroplasma isolates and ferroplasma acidarmanus sp. nov., extreme acidophiles from acid mine drainage and industrial bioleaching environments. *Appl. environmental microbiology* **70**, 2079–2088 (2004).
18. Perevalova, A. A. *et al.* Fervidicoccus fontis gen. nov., sp. nov., an anaerobic, thermophilic crenarchaeote from terrestrial hot springs, and proposal of fervidicoccaceae fam. nov. and fervidicoccales ord. nov. *Int. journal systematic evolutionary microbiology* **60**, 2082–2088 (2010).
19. Roh, S. W. *et al.* Halalkalicoccus jeotgali sp. nov., a halophilic archaeon from shrimp jeotgal, a traditional korean fermented seafood. *Int. journal systematic evolutionary microbiology* **57**, 2296–2298 (2007).
20. Juez, G., Rodriguez-Valera, F., Ventosa, A. & Kushner, D. J. Haloarcula hispanica spec. nov. and haloferax gibbonsii spec, nov., two new species of extremely halophilic archaebacteria. *Syst. Appl. Microbiol.* **8**, 75–79 (1986).
21. Robinson, J. L. *et al.* Growth kinetics of extremely halophilic archaea (family halobacteriaceae) as revealed by arrhenius plots. *J. bacteriology* **187**, 923–929 (2005).
22. Tindall, B. J. *et al.* Complete genome sequence of halomicrobium mukohataei type strain (arg-2 t). *Standards Genomic Sci.* **1**, 270 (2009).
23. Anderson, I. *et al.* Complete genome sequence of halopiger xanaduensis type strain (sh-6 t). *Standards genomic sciences* **6**, 31 (2012).
24. Burns, D. G. *et al.* Haloquadratum walsbyi gen. nov., sp. nov., the square haloarchaeon of walsby, isolated from saltern crystallizers in australia and spain. *Int. J. Syst. Evol. Microbiol.* **57**, 387–392 (2007).

25. Franzmann, P. *et al.* Halobacterium lacusprofundi sp. nov., a halophilic bacterium isolated from deep lake, antarctica. *Syst. Appl. Microbiol.* **11**, 20–27 (1988).

26. Castillo, A. *et al.* Halovivax ruber sp. nov., an extremely halophilic archaeon isolated from lake xilinhot, inner mongolia, china. *Int. journal systematic evolutionary microbiology* **57**, 1024–1027 (2007).

27. Jahn, U. *et al.* Nanoarchaeum equitans and ignicoccus hospitalis: new insights into a unique, intimate association of two archaea. *J. bacteriology* **190**, 1743–1750 (2008).

28. Niederberger, T. D., Götz, D. K., McDonald, I. R., Ronimus, R. S. & Morgan, H. W. Ignisphaera aggregans gen. nov., sp. nov., a novel hyperthermophilic crenarchaeote isolated from hot springs in rotorua and tokaanu, new zealand. *Int. journal systematic evolutionary microbiology* **56**, 965–971 (2006).

29. Liu, L.-J., You, X.-Y., Guo, X., Liu, S.-J. & Jiang, C.-Y. Metallosphaera cuprina sp. nov., an acidothermophilic, metal-mobilizing archaeon. *Int. journal systematic evolutionary microbiology* **61**, 2395–2400 (2011).

30. Han, C. J., Park, S. H. & Kelly, R. M. Acquired thermotolerance and stressed-phase growth of the extremely thermoacidophilic archaeon metallosphaera sedula in continuous culture. *Appl. environmental microbiology* **63**, 2391–2396 (1997).

31. MacDonald, D. The encyclopedia of mammals. equinox ltd (1984).

32. Miller, T. L., Wolin, M., de Macario, E. C. & Macario, A. Isolation of methanobrevibacter smithii from human feces. *Appl. Environ. Microbiol.* **43**, 227–232 (1982).

33. Jeanthon, C. *et al.* Methanococcus infernus sp. nov., a novel hyperthermophilic lithotrophic methanogen isolated from a deep-sea hydrothermal vent. *Int. J. Syst. Evol. Microbiol.* **48**, 913–919 (1998).

34. Jones, W., Leigh, J. A., Mayer, F., Woese, C. & Wolfe, R. Methanococcus jannaschii sp. nov., an extremely thermophilic methanogen from a submarine hydrothermal vent. *Arch. Microbiol.* **136**, 254–261 (1983).

35. Jeanthon, C. *et al.* Methanococcus vulcanius sp. nov., a novel hyperthermophilic methanogen isolated from east pacific rise, and identification of methanococcus sp. dsm 4213tas methanococcus fervens sp. nov. *Int. J. Syst. Evol. Microbiol.* **49**, 583–589 (1999).

36. Sakai, S., Conrad, R., Liesack, W. & Imachi, H. Methanocella arvoryzae sp. nov., a hydrogenotrophic methanogen isolated from rice field soil. *Int. journal systematic evolutionary microbiology* **60**, 2918–2923 (2010).

37. Lü, Z. & Lu, Y. Methanocella conradii sp. nov., a thermophilic, obligate hydrogenotrophic methanogen, isolated from chinese rice field soil. *PloS one* **7**, e35279 (2012).

38. Sakai, S. *et al.* Methanocella paludicola gen. nov., sp. nov., a methane-producing archaeon, the first isolate of the lineage 'rice cluster i', and proposal of the new archaeal order methanocellales ord. nov. *Int. J. Syst. Evol. Microbiol.* **58**, 929–936 (2008).

39. Jones, W. J., Paynter, M. & Gupta, R. Characterization of methanococcus maripaludis sp. nov., a new methanogen isolated from salt marsh sediment. *Arch. microbiology* **135**, 91–97 (1983).

40. Whitman, W., Ankwanda, E. & Wolfe, R. Nutrition and carbon metabolism of methanococcus voltae. *J. Bacteriol.* **149**, 852–863 (1982).

41. Anderson, I. J. *et al.* Complete genome sequence of methanoculleus marisnigri romesser et al. 1981 type strain jr1. *Standards Genomic Sci.* **1**, 189 (2009).

42. Bowers, K. J. & Wiegel, J. Temperature and ph optima of extremely halophilic archaea: a mini-review. *Extrem.* **15**, 119–128 (2011).

43. Paterek, J. R. & Smith, P. H. Methanohalophilus mahii gen. nov., sp. nov., a methylotrophic halophilic methanogen†. *Int. J. Syst. Evol. Microbiol.* **38**, 122–123 (1988).

44. Zhang, G., Jiang, N., Liu, X. & Dong, X. Methanogenesis from methanol at low temperatures by a novel psychrophilic methanogen,"methanolobus psychrophilus" sp. nov., prevalent in zoige wetland of the tibetan plateau. *Appl. environmental microbiology* **74**, 6114–6120 (2008).

45. Lomans, B. P. *et al.* Isolation and characterization of methanomethylovorans hollandica gen. nov., sp. nov., isolated from freshwater sediment, a methylotrophic methanogen able to grow on dimethyl sulfide and methanethiol. *Appl. environmental microbiology* **65**, 3641–3650 (1999).

46. Yashiro, Y. *et al.* Methanoregula formicica sp. nov., a methane-producing archaeon isolated from methanogenic sludge. *Int. journal systematic evolutionary microbiology* **61**, 53–59 (2011).

47. PATEL, G. B. & SPROTT, G. D. Methanosaeta concilii gen. nov., sp. nov.("methanothrix concilii") and methanosaeta thermoacetophila nom. rev., comb. nov.†. *Int. J. Syst. Evol. Microbiol.* **40**, 79–82 (1990).

48. Ma, K., Liu, X. & Dong, X. Methanosaeta harundinacea sp. nov., a novel acetate-scavenging methanogen isolated from a uasb reactor. *Int. journal systematic evolutionary microbiology* **56**, 127–131 (2006).

49. Mathrani, I. M., Boone, D. R., Mah, R. A., Fox, G. E. & Lau, P. P. Methanohalophilus zhilinae sp. nov., an alkaliphilic, halophilic, methylotrophic methanogen. *Int. J. Syst. Evol. Microbiol.* **38**, 139–142 (1988).

50. Gunnigle, E. *et al.* A functional approach to uncover the low-temperature adaptation strategies of the archaeon

methanosarcina barkeri. *Appl. environmental microbiology* **79**, 4210–4219 (2013).

51. Maestrojuan, G. M. & Boone, D. R. Characterization of methanosarcina barkeri mst and 227, methanosarcina mazei s-6t, and methanosarcina vacuolata z-761t. *Int. J. Syst. Evol. Microbiol.* **41**, 267–274 (1991).

52. Ding, X. *et al.* Isolation and characterization of a new strain of methanothermobacter marburgensis dx01 from hot springs in china. *Anaerobe* **16**, 54–59 (2010).

53. Takai, K., Inoue, A. & Horikoshi, K. Methanothermococcus okinawensis sp. nov., a thermophilic, methane-producing archaeon isolated from a western pacific deep-sea hydrothermal vent system. *Int. journal systematic evolutionary microbiology* **52**, 1089–1095 (2002).

54. Stetter, K. O. *et al.* Methanothermus fervidus, sp. nov., a novel extremely thermophilic methanogen isolated from an icelandic hot spring. *Zentralblatt für Bakteriologie Mikrobiologie und Hyg. I. Abt. Orig. C: Allgemeine, angewandte und ökologische Mikrobiologie* **2**, 166–178 (1981).

55. Burggraf, S. *et al.* Methanococcus igneus sp. nov., a novel hyperthermophilic methanogen from a shallow submarine hydrothermal system. *Syst. Appl. Microbiol.* **13**, 263–269 (1990).

56. Burns, D. G. *et al.* Natronomonas moolapensis sp. nov., non-alkaliphilic isolates recovered from a solar saltern crystallizer pond, and emended description of the genus natronomonas. *Int. journal systematic evolutionary microbiology* **60**, 1173–1176 (2010).

57. Völkl, P. *et al.* Pyrobaculum aerophilum sp. nov., a novel nitrate-reducing hyperthermophilic archaeum. *Appl. Environ. Microbiol.* **59**, 2918–2926 (1993).

58. Huber, R., Sacher, M., Vollmann, A., Huber, H. & Rose, D. Respiration of arsenate and selenate by hyperthermophilic archaea. *Syst. Appl. Microbiol.* **23**, 305–314 (2000).

59. Sako, Y., Nunoura, T. & Uchida, A. Pyrobaculum oguniense sp. nov., a novel facultatively aerobic and hyperthermophilic archaeon growing at up to 97 degrees c. *Int. journal systematic evolutionary microbiology* **51**, 303–309 (2001).

60. Erauso, G. *et al.* Pyrococcus abyssi sp. nov., a new hyperthermophilic archaeon isolated from a deep-sea hydrothermal vent. *Arch. Microbiol.* **160**, 338–349 (1993).

61. Fiala, G. & Stetter, K. O. Pyrococcus furiosus sp. nov. represents a novel genus of marine heterotrophic archaebacteria growing optimally at 100 c. *Arch. Microbiol.* **145**, 56–61 (1986).

62. Birrien, J.-L. *et al.* Pyrococcus yayanosii sp. nov., an obligate piezophilic hyperthermophilic archaeon isolated from a deep-sea hydrothermal vent. *Int. journal systematic evolutionary microbiology* **61**, 2827–2881 (2011).

63. Blöchl, E. *et al.* Pyrolobus fumarii, gen. and sp. nov., represents a novel group of archaea, extending the upper temperature limit for life to 113 c. *Extrem.* **1**, 14–21 (1997).

64. Arab, H., Völker, H. & Thomm, M. Thermococcus aegaeicus sp. nov. and staphylothermus hellenicus sp. nov., two novel hyperthermophilic archaea isolated from geothermally heated vents off palaeochori bay, milos, greece. *Int. journal systematic evolutionary microbiology* **50**, 2101–2108 (2000).

65. Chen, L. *et al.* The genome of sulfolobus acidocaldarius, a model organism of the crenarchaeota. *J. bacteriology* **187**, 4992–4999 (2005).

66. Jaubert, C. *et al.* Genomics and genetics of sulfolobus islandicus lal14/1, a model hyperthermophilic archaeon. *Open biology* **3**, 130010 (2013).

67. She, Q. *et al.* The complete genome of the crenarchaeon sulfolobus solfataricus p2. *Proc. Natl. Acad. Sci.* **98**, 7835–7840 (2001).

68. Marteinsson, V. T. *et al.* Thermococcus barophilus sp. nov., a new barophilic and hyperthermophilic archaeon isolated under high hydrostatic pressure from a deep-sea hydrothermal vent. *Int. J. Syst. Evol. Microbiol.* **49**, 351–359 (1999).

69. Jolivet, E., L'Haridon, S., Corre, E., Forterre, P. & Prieur, D. Thermococcus gammatolerans sp. nov., a hyperthermophilic archaeon from a deep-sea hydrothermal vent that resists ionizing radiation. *Int. J. Syst. Evol. Microbiol.* **53**, 847–851 (2003).

70. Atomi, H., Fukui, T., Kanai, T., Morikawa, M. & Imanaka, T. Description of thermococcus kodakaraensis sp. nov., a well studied hyperthermophilic archaeon previously reported as pyrococcus sp. kod1. *Archaea* **1**, 263–267 (2004).

71. Seob, B. S. *et al.* Thermococcus onnurineus sp. nov., a hyperthermophilic archaeon isolated from a deep-sea hydrothermal vent area at the pacmanus field. *J. microbiology biotechnology* **16**, 1826–1831 (2006).

72. Miroshnichenko, M. L. *et al.* Isolation and characterization of thermococcus sibiricus sp. nov. from a western siberia high-temperature oil reservoir. *Extrem.* **5**, 85–91 (2001).

73. Zillig, W. *et al.* The archaebacterium thermofilum pendens represents, a novel genus of the thermophilic, anaerobic sulfur respiring thermoproteales. *Syst. Appl. Microbiol.* **4**, 79–87 (1983).

74. Mardanov, A. V. *et al.* Complete genome sequence of the hyperthermophilic cellulolytic crenarchaeon "thermogladius cellulolyticus" 1633. *J. bacteriology* **194**, 4446–4447 (2012).

75. Bonch-Osmolovskaya, E., Miroshnichenko, M., Kostrikina, N., Chernych, N. & Zavarzin, G. Thermoproteus

uzoniensis sp. nov., a new extremely thermophilic archaebacterium from kamchatka continental hot springs. *Arch. microbiology* **154**, 556–559 (1990).

76. Huber, R., Dyba, D., Huber, H., Burggraf, S. & Rachel, R. Sulfur-inhibited thermosphaera aggregans sp. nov., a new genus of hyperthermophilic archaea isolated after its prediction from environmentally derived 16s rrna sequences. *Int. J. Syst. Evol. Microbiol.* **48**, 31–38 (1998).

77. Itoh, T., Suzuki, K.-i. & Nakase, T. Vulcanisaeta distributa gen. nov., sp. nov., and vulcanisaeta souniana sp. nov., novel hyperthermophilic, rod-shaped crenarchaeotes isolated from hot springs in japan. *Int. journal systematic evolutionary microbiology* **52**, 1097–1104 (2002).

78. Gumerov, V. M. *et al.* Complete genome sequence of "vulcanisaeta moutnovskia" strain 768-28, a novel member of the hyperthermophilic crenarchaeal genus vulcanisaeta. *J. bacteriology* **193**, 2355–2356 (2011).
